# On the Sensing and Calibration of Residual Stresses Measurements in the Incremental Hole-Drilling Method

**DOI:** 10.3390/s21227447

**Published:** 2021-11-09

**Authors:** Mohamed M. A. Ammar, Bijan Shirinzadeh, Kai Zhong Lai, Weichen Wei

**Affiliations:** 1 Robotics and Mechatronics Research Laboratory, Department of Mechanical and Aerospace Engineering, Monash University, Clayton 3800, Australia; 2 Department of Mechanical Engineering, Faculty of Engineering, Alexandria University, Alexandria 11432, Egypt

**Keywords:** residual stresses, incremental hole-drilling, finite element modeling (fem), robotic fiber placement (RFP)

## Abstract

The current study presents three calibration approaches for the hole-drilling method (HDM). A total of 72 finite element models and 144 simulations were established to calibrate the measurements of the strain sensors. The first approach assumed the stresses acted on the boundaries of the drilled hole and thus analyzed the surrounding displacements field. The second analysis considered the loads on the outer surfaces of the specimen while measuring the strains’ differences between the model with and without the drilled hole. The third approach was more comprehensive as it considered the mechanical and thermal effects of the drilling operations. The proposed approaches were applied to two different materials (AISI 1045 and CFRP). The steel specimens were machined using a CNC lathe while the composite laminates were manufactured using the robotic fiber placement (RFP) process. Subsequently, the residual stresses (RSs) were measured using the HDM. The obtained data were compared with X-ray diffraction measurements for validation. The results showed better estimation of the RSs when utilizing the third approach and clear underestimation of the stresses using the second approach. A divergence in RSs values between the three approaches was also detected when measuring the stresses in the internal layers of the composite laminates.

## 1. Introduction

Manufacturing and processing materials are considered the main sources of internal stresses (i.e., residual stresses (RSs)) inside structures. The induced RSs are one of the most important parameters of surface integrity, as they can affect the quality of the final products. The fatigue life, brittle fracture process, dimensional stability, distortion, and corrosion resistance can all be considerably impacted by RSs. In addition, RSs can be manipulated to improve material behavior under specific mechanical applications [1]. Thus, much research has been conducted in order to measure and predict the induced RSs inside materials, as this is considered an important stage for designing the structural components and estimating their reliability [2,3,4,5]. The stresses inside the material are usually estimated in two common ways, optical methods [6,7,8,9] or using physical sensors [10,11,12]. Different experimental and numerical techniques are utilized to precisely determine the magnitude and types of the stresses, such as the hole-drilling method (HDM) [13,14], X-ray diffraction (XRD) [15], the neutron diffraction method [16], the slitting method [17], and the curvature method [18].

Although progress has been achieved in developing measurement techniques for RSs, more effort is still required to establish cost-effective, efficient, and precise technologies for the benefit of analysis and redistribution of RSs. The measurement techniques are categorized based on their effect on the structure into three main groups, nondestructive, semi-destructive, and destructive techniques. The nondestructive methods, which depend on analyzing material parameters related to the stresses such as diffraction techniques, are commonly used for measurement of stresses inside metals. However, these technologies are not able to precisely estimate the stresses inside composites due to their microcrystalline or amorphous nature. Therefore, mechanical methods, that mainly rely on material stress relaxation to measure the stresses, are introduced for orthotropic materials. Among all the mechanical methods, the HDM is one of the most commonly utilized semi-destructive measurement methods. Its relative simplicity, reliability, and good accuracy set it apart from other available techniques. Additionally, the HDM has been developed into the incremental hole-drilling method (IHDM) to measure the stresses inside composites by adding an incremental behavior to the process [19,20].

Implementing the HDM includes three steps; first, experimental measurements of the released strains; second, calibration of those measurements; and third, mathematical calculation of the RSs. The calibration process is an important step to obtain reliable results, as it considers the effect of all the experimental conditions such as sample geometry, hole geometry, and material type and conditions. Several experimentally evaluated calibration coefficients were determined in the HDM standard (E 837-08) [21] to perform the calibration process. However, these coefficients were good only for a particular type of material with particular experimental parameters. Blodorn et al. [22] determined the calibration coefficients numerically using finite element modeling (FEM). A blind hole was created in the workpiece, and the load was applied to trigger the element displacement and determine the coefficients. Uniform RSs measurements were obtained inside the A36 steel, AISI304L stainless steel, and AA6061 aluminum alloy. Significant differences were detected between the obtained coefficients and the standard (i.e., experimentally evaluated) values. Different finite element approaches were reported in [1,23,24,25], including subjecting the loads on the internal surfaces of the created hole, and in [20,22,26], considering loads that act on the external borders of the specimens. Although several approaches have been implemented to find the coefficient numerically, there was no validation or confirmation of the precision of the measured stresses.

To the best of the authors’ knowledge, there has been no additional effort to improve the calibration process, especially to obtain a better estimation of RSs inside composite materials. The current work provides a complete study of the IHDM. The experimental measurements, theoretical approach, and numerical calibration are studied to understand the RSs measurement technique. Further, the machining process [27] of the metal specimens and the automated manufacturing [28,29] of the composite parts are described in this work. The contributions of the present research are as follows:

- Introducing three different approaches to calibrate the strain sensors’ measurements of RSs using the IHDM. The proposed processes were deployed through 72 models on two different materials (i.e., isotropic and orthotropic);
- Validating the proposed approaches by comparing them to XRD measurements of machined metal samples (i.e., AISI 1045);
- Investigating the differences between the approaches when estimating the in-depth RSs inside orthotropic materials (i.e., carbon fiber reinforced polymers (CFRP));
- Studying the characteristics of the accuracy of the approaches for the isotropic and orthotropic materials.

## 2. Theory of the Hole-Drilling Method (HDM)

The HDM is a popular and effective approach for estimating the uniform and nonuniform RSs inside the materials. This technique has the potential to measure the in-depth RSs with high accuracy. A series of experimental, numerical, and analytical approaches were developed and combined to make this technique suitable for measuring the in-depth stresses in composites and metal structures. This method is attractive for measuring RSs, as it possesses many advantages, such as simple setup and less material destruction. Additionally, it has the ability to measure the stresses inside amorphous material and at higher depths compared with diffraction techniques. This technique was initially applied to homogeneous and isotropic materials. Subsequently, it was developed and adapted to measure the stresses of orthotropic materials by the addition of the calibration methodology. The calibration technique was used to correct the measured strains and calculate the stresses by estimating the number of coefficients numerically or experimentally.

**Figure 1:**
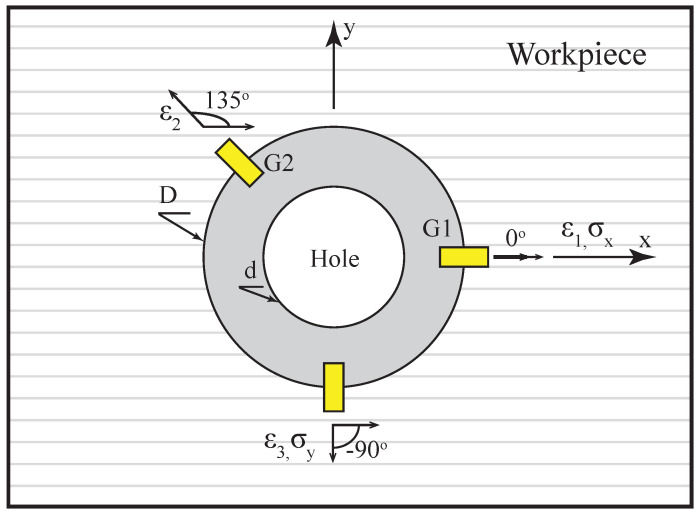
Typical configuration of 45${}^{\circ }$ rosette strain gauge.

According to the ASTM E837-13a standard [21], this technique considers the released stresses when incrementally drilling a small hole in the material. Removing each layer triggers the materiel to establish a new equilibrium condition. The hole geometry changes due to the movement of the hole borders towards the equilibrium position, corresponding to each removed layer and the change in the surrounding constraints on the hole. The material deformations are transformed to represent the differences in the directional strains relative to the undisturbed condition. These strains are measured by strain gauges in different directions to completely detect all the material movements caused by the previously existing stresses. The most common type of strain gauge utilized for this purpose is the rosette strain gauge. The gauges are usually bonded to the surface of the structure, in the area of interest; subsequently, the hole is drilled exactly in the geometrical center of the configuration of the gauges. The profile of the RSs through the thickness can then be determined as a result of the developed strains. Figure 1 shows the 45${}^{\circ }$ rosette strain gauge, which is commonly used for composite applications and was applied in this work. The first gauge (G1) is always aligned with the positive X-direction (i.e., the main fiber direction in the case of composites) and with angles 135${}^{\circ }$ and −90${}^{\circ }$ for the second and third gauges (G2 and G3), respectively.

In Figure 1, ϵ(1), ϵ(2), and ϵ(3) represent the total measured strains on the top of the specimen in three directions, and $\sigma_{x}$ and $\sigma_{y}$ are the in-plane stress components. The hole diameter is denoted by *d*, and the diameter of the gauge center is *D*.

The accuracy of the RSs measurements through the HDM may be affected by several parameters. This includes the geometry of the strain gauges, the ratio between the size of the gauge and the size of the hole (D/d), the ratio between the specimen thickness and the gauge size, the eccentricity between the hole center and the center of the strain gauges’ configuration, drilling speed, and the feed-speeds. Additionally, the drilling speed, feed rate, and the time of the continuous drilling, which is related to the thickness of each increment, have a significant effect on the measured strains for two reasons: first, the generation of heat during this process; and second, the existence of microcracks. Thus, an increase in the relaxation strains might be detected, which leads to overestimation of the RSs. Therefore, these parameters should be carefully adjusted and optimized to minimize their effects. Another possible solution to address this issue is to consider the effect of these conditions in the calibration process.

### 2.1. Analytical Formulation

This section presents the analytical approach that was applied to the measured strains along with the calibration process based on the finite element analysis (FEA) technique in order to determine the RSs. The current theory was developed to consider the incremental behavior of drilling the hole along with the change in the stresses’ states after each increment.

The surface strains can be written as a function of the in-plane principal stresses as shown in Equation (1). This is a radial strain at a predefined direction and known distance from the hole center. In this approach, the change of the stress component in the third direction normal to surface $\sigma_{z}$ is assumed to be negligible compared to the in-plane stresses [24,30].

(1) $$\epsilon \left(\psi \right)=A\left(\sigma_{1}+\sigma_{2}\right)+\left(\sigma_{1}-\sigma_{2}\right)\left(B\cos\left(2\psi \right)+C\sin\left(2\psi \right)\right)$$

where ϵ(ψ) is the change of the strain at direction ψ of the maximum principal stress from the X-direction (i.e., the first gauge), while $\sigma_{1}$ and $\sigma_{2}$ represent the principal stresses calculated in each increment, and the three coefficients *A*, *B*, and *C* are determined through FEM. The current technique depends on measuring the strains in three directions around the drilled hole. The stresses are then calculated in any direction based on these strains with the aid of the numerical process.

At any drilling increment *i*, the material deformations are measured and the corresponding strains in three directions can be represented by the Equations (2)–(4).

(2) $$\epsilon_{i}^{1}=A_{i}\left(\sigma_{1i}+\sigma_{2i}\right)+\left(\sigma_{1i}-\sigma_{2i}\right)\left(B_{i}\cos\left(2\psi_{i}\right)+C_{i}\sin\left(2\psi_{i}\right)\right)$$

(3) $$\epsilon_{i}^{2}=A_{i}\left(\sigma_{1i}+\sigma_{2i}\right)+\left(\sigma_{1i}-\sigma_{2i}\right)\left(B_{i}\cos\left(2\left(\psi_{i}+\alpha \right)\right)+C_{i}\sin\left(2\left(\psi_{i}+\alpha \right)\right)\right)$$

(4) $$\epsilon_{i}^{3}=A_{i}\left(\sigma_{1i}+\sigma_{2i}\right)+\left(\sigma_{1i}-\sigma_{2i}\right)\left(B_{i}\cos\left(2\left(\psi_{i}+\beta \right)\right)+C_{i}\sin\left(2\left(\psi_{i}+\beta \right)\right)\right)$$

where $\epsilon_{i}^{1}$, $\epsilon_{i}^{2}$, and $\epsilon_{i}^{3}$ are the incremental strains in three directions of the three gauges included in the configuration of the rosette type. The second and the third strain gauges are located at angles α and β from the reference strain gauge (X-direction), respectively.

The main limitation to this approach is the determination of the exact strains developed due to drilling a specific increment, and exclusion of the strains developed due to the contribution of the previously removed layers. Therefore, except for the first increment, the strain measured on the surface is a combination of the effect of drilling an increment and the effect of the formerly removed layers. Thus, the formulas used to calculate the in-plane strains were adapted to consider these effects, as expressed in Equations (5)–(7).

(5) $$\epsilon_{i}^{1}=\epsilon_{ti}^{1}-\sum_{k=1}^{i-1}\epsilon_{ki}^{1}$$

(6) $$\epsilon_{i}^{2}=\epsilon_{ti}^{2}-\sum_{k=1}^{i-1}\epsilon_{ki}^{2}$$

(7) $$\epsilon_{i}^{3}=\epsilon_{ti}^{3}-\sum_{k=1}^{i-1}\epsilon_{ki}^{3}$$

where $\epsilon_{ti}^{1}$, $\epsilon_{ti}^{2}$, and $\epsilon_{ti}^{3}$ refer to the directional strains measured on the surface of the structure by the gauges when drilling a specific increment *i*. The contributions of the former removed increment *k* to the measured strains of the current layer $i^{th}$ are represented by $\epsilon_{ki}^{1}$, $\epsilon_{ki}^{2}$, and $\epsilon_{ki}^{3}$. The values of these strains’ contributions are defined according to Equations (8)–(10).

(8) $$\epsilon_{ki}^{1}=A_{kn}\left(\sigma_{1ki}+\sigma_{2ki}\right)+\left(\sigma_{1ki}-\sigma_{2ki}\right)\left(B_{ki}\cos\left(2\psi_{k}\right)+C_{ki}\sin\left(2\psi_{i}\right)\right)$$

(9) $$\epsilon_{ki}^{2}=A_{ki}\left(\sigma_{1ki}+\sigma_{2ki}\right)+\left(\sigma_{1ki}-\sigma_{2ki}\right)\left(B_{ki}\cos\left(2\left(\psi_{k}+\alpha \right)\right)+C_{ki}\sin\left(2\left(\psi_{k}+\alpha \right)\right)\right)$$

(10) $$\epsilon_{ki}^{3}=A_{ki}\left(\sigma_{1ki}+\sigma_{2ki}\right)+\left(\sigma_{1ki}-\sigma_{2ki}\right)\left(B_{ki}\cos\left(2\left(\psi_{k}+\beta \right)\right)+C_{ki}\sin\left(2\left(\psi_{k}+\beta \right)\right)\right)$$

**Figure 2:**
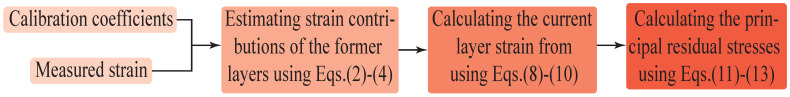
Flowchart of HDM’s analytical formulation.

In these relationships, the terms $\sigma_{1ki}$ and $\sigma_{2ki}$ represent the principal stresses that have already been calculated from the last increment. On the other hand, the Equations (8)–(10) include a set of three calibration coefficients $A_{ki}$, $B_{ki}$, and $C_{ki}$, and as mentioned earlier, they will be determined through the FEA. Finally, the principal stresses at each layer are obtained by the Equations (11)–(13). Figure 2 shows the entire process of estimating the RSs. Equations (11)–(13) are based on involving the rosette strain gauges of 45°, and these equations would be changed according to the utilized configuration of the strain gauges.

(11) $$\sigma_{1i}=\frac{\epsilon_{i}^{1}\left(A_{i}-B_{i}\sin\left(2\psi_{i}\right)+C_{i}\cos\left(2\psi_{i}\right)\right)-\epsilon_{in}^{2}\left(A_{i}-B_{i}\cos\left(2\psi_{i}\right)-C_{i}\sin\left(2\psi_{i}\right)\right)}{2A_{i}B_{i}\left(-\sin\left(2\psi_{i}\right)+\cos\left(2\psi_{i}\right)\right)+2A_{i}C_{i}\left(\sin\left(2\psi_{i}\right)+\cos\left(2\psi_{i}\right)\right)}$$

(12) $$\sigma_{2i}=\frac{-\epsilon_{i}^{1}\left(A_{i}+B_{i}\sin\left(2\psi_{i}\right)-C_{i}\cos\left(2\psi_{i}\right)\right)+\epsilon_{i}^{2}\left(A_{i}+B_{i}\cos\left(2\psi_{i}\right)+C_{i}\sin\left(2\psi_{i}\right)\right)}{2A_{i}B_{i}\left(-\sin\left(2\psi_{i}\right)+\cos\left(2\psi_{i}\right)\right)+2A_{i}C_{i}\left(\sin\left(2\psi_{i}\right)+\cos\left(2\psi_{i}\right)\right)}$$

(13) $$\psi_{i}=\frac{1}{2}\tan^{-1}\left[\frac{C_{i}\left(\epsilon_{i}^{3}-\epsilon_{i}^{1}\right)-B_{i}\left(2\epsilon_{i}^{2}-\epsilon_{i}^{1}-\epsilon_{i}^{3}\right)}{C_{i}\left(2\epsilon_{i}^{2}-\epsilon_{i}^{1}-\epsilon_{i}^{3}\right)+B_{i}\left(\epsilon_{i}^{3}-\epsilon_{i}^{1}\right)}\right]$$

Since we know the magnitudes and the directions of the principal stresses, they can be resolved into X- and Y-directions to find the RSs in these two specific directions (i.e., parallel and normal to the direction of the reference strain gauge).

## 3. Numerical Modeling of the Calibration Process

Finite element simulations have been utilized in several studies [22,30] and have provided reliable determinations of the calibration coefficients. The superposition approach has shown good estimation in most of these attempts, by considering the main strains of the drilled layer along with the effect of the removed layer, as explained in Section 2. This approach showed that the calculation of the strain relaxation and the determination of the tabulated coefficients could be achieved by creating a stress field in the area of interest, which surrounds the hole. When the stresses acted in the locations of the strain gauges, the elements on the specimen surface moved, and the average element displacements were measured for each gauge. A stepwise stress field was applied to each layer, and the displacements on the surface were analyzed to define the relationship between the drilling of each increment and the resulting strains. The sets of the coefficients were determined by considering the average difference between the displacements at the border of each gauge. The strain over the gauge length was calculated by the displacement difference between the gauge borders. Subsequently, the first tabulated coefficient *A* was determined by applying identical biaxial stress in each increment in directions X and Y. Equation (14) was applied to calculate all the values of the variable $A_{ki}$.

(14) $$A_{ki}=\frac{U_{ki}\left(R_{2},\psi_{i}=0\right)-U_{ki}\left(R_{1},\psi_{i}=0\right)}{2\sigma L},L=R_{2}-R_{1}$$

where $U_{ki}$ refers to the radial displacement (deformation) obtained by FEA at the strain gauge edges $R_{1}$ and $R_{2}$. Therefore, *L* is a representation of the gauge length. The applied stress field is also considered in the formula by the symbol σ. Thus, eventually, the coefficient $A_{ii}$ is a result of the strain developed in layer *i* when applying the stress on the same layer, while $A_{ki}$ is a result of the strain developed in layer *i* when applying the stress on the former layer *k*. More details on the calculation procedures of the calibration coefficient can be found in [22].

On the other hand, combined stresses (normal and shear stresses) with the magnitudes +σcos(2ψ) and −σsin(2θ), respectively, were applied to each increment to evaluate the two coefficients $B_{ki}$ and $C_{ki}$. The implementation of biaxial stresses in the case of the three coefficients will be described in detail for each of the constructed models. Consequently, the two coefficients, $B_{ki}$ and $C_{ki}$, were obtained according to the Equations (15) and (16), respectively.

(15) $$B_{ki}=\frac{U_{ki}\left(R_{2},\psi_{i}=\frac{\pi }{4}\right)-U_{ki}\left(R_{1},\psi_{i}=\frac{\pi }{4}\right)}{2\sigma L}$$

(16) $$C_{ki}=\frac{U_{ki}\left(R_{2},\psi_{i}=\frac{\pi }{2}\right)-U_{ki}\left(R_{1},\psi_{i}=\frac{\pi }{2}\right)}{2\sigma L}$$

After obtaining the set of the three variables in each increment, Equations (8)–(13) used these values to obtain the RSs. The coefficients were calculated and remained constants for the entire test for the same sample. In order to create the required stresses and determine the resultant strains, three different approaches were constructed in the present work. Each approach was represented by composite and metal models, while the models were built using the Abaqus software package. The models were constructed through Abaqus/standard with three-dimensional frameworks. One quarter of each specimen was analyzed due to the similarity of the model in the X- and Y-directions. The elements’ edge size in all the models was established approximately to 0.05 mm in the area surrounding the hole, which may experience stress concentration and increased progressively outward to the edges of the specimens. In each approach, two different models were established: first, the square specimen model for the composite part; second, the circular specimen for the metal part. The details of these approaches were as follows.

### 3.1. Internal Stress Field Approach (IHDM-1)

**Figure 3:**
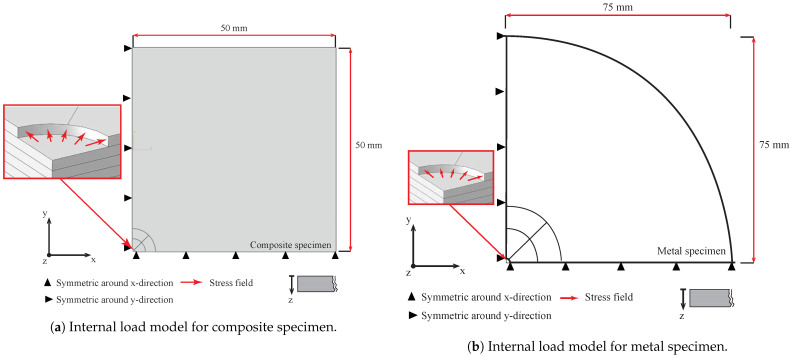
Internal load models.

In this section, the basic finite element approach used to calibrate the strain measurements is described. A linear elastic analysis was utilized in the corresponding models. The stresses in the created models were applied to the internal surfaces of the drilled hole. The 8-node elements (C3D8) were utilized in these analyses. Sixteen simulations were conducted for each model to obtain the required coefficients. A new set of elements that represented the following drill increment in the hole was removed after each simulation. Each model was performed twice; the first run was to obtain coefficient *A*, and the second was to obtain coefficients *B* and *C*. All the other faces of the workpiece along with the far borders were left unstressed. Figure 3a,b shows the constructed model for the square composite specimen and the cylindrical metal specimen, respectively. The applied loads and boundary conditions are presented in the figures. The symmetrical boundary conditions were considered by defining zero velocities in X- and Y-directions of the relevant surfaces. The normal and shear RSs were assumed to act in planes parallel to the workpiece surface. All the stresses in the direction normal to the surface were assumed to be very small and their effect on the surface strains was neglected. The model geometry was similar to the actual samples, as will be explained in Section 4.3. In the case of determining the coefficient $A_{ki}$, a uniform pressure acting on the internal surface of the hole for each increment was the source of the stress field at the locations of the strain gauges. Separate models were required to determine the other two coefficients $B_{ki}$ and $C_{ki}$. Normal stress equal to +σcos2θ and shear stress equal to −σsin2θ should act on the hole surface. These loads were applied to the workpiece by creating a software module (DLOAD), which was combined with the Abaqus models. The models’ analysis was performed based on one static general step to apply the loads. All the surfaces of the workpieces in these models were assumed adiabatic. The strain measurements were collected from the locations of each strain gauge. Stresses with unity values were substituted in Equations (14)–(16) to obtain the relevant coefficients.

### 3.2. External Stress Field Approach (IHDM-2)

**Figure 4:**
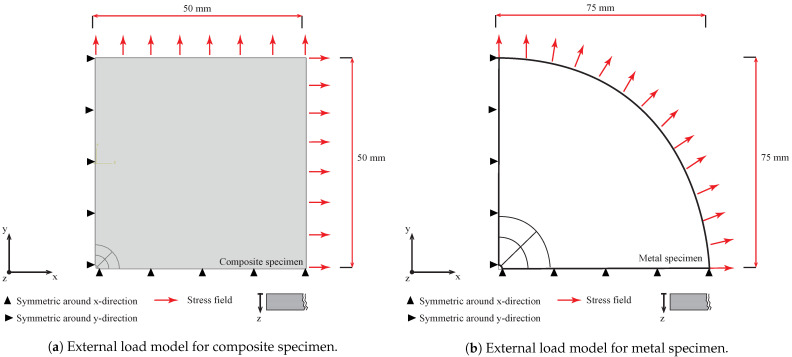
External load models.

Opposite to the first approach, the current models included specimens with external loads. Several methods close to this approach have been conducted in former research work for isotropic material such as [22]. In the present work, the stress field was applied to the external surfaces of the specimen. Each increment had two related models; the first model did not include a hole as the specimen was built as a completely solid part, while the second model possessed a pre-existing hole. In both models, the stresses acted on the outer border of the specimen for each increment as shown in Figure 4. A biaxial load of 1 MPa was applied on the external surface of each increment in X- and Y-directions to obtain factor *A*. In addition, factors *B* and *C* were determined by applying a load of 1 MPa positive in the X-direction and negative in the Y-direction. The strain field for each gauge was calculated by the difference of the strains from the specimen without a hole and the specimen with a blind hole. Therefore, the equivalent stresses were defined by the subtraction of the average stresses in the area around the hole for the specimen with the hole from that of the specimen without the blind hole. The loads were applied incrementally similar to the internal stress approach; however, the loads here act on the outer incremental surfaces. The analyses were performed based on one loading step by considering only the elastic behavior of the material. Up to 72 simulations were completed in total for both metal and composite models.

### 3.3. Coupled Thermal-Mechanical Approach (IHDM-3)

The proposed approach considered two simultaneous loads acting on the specimen, mechanical stresses and heating effects. The drilling process is accompanied by generation of heat, which increases the specimen temperature around the hole borders. The specimen heating may cause inelastic deformation of the material, which could affect the reading of the strain gauges. In the current experiments, the material was left for a couple of minutes to cool from the drilling effect. Thus, specimen heating and the subsequent cooling should be considered in the calibration process. In order to analyze these effects, a coupled temperature-displacement model was assumed. Temperature-dependent material properties were adopted, as detailed in Section 4. This approach was based on two subsequent simulation steps. The first step included applying the stresses and the heat effects on the internal surface of the hole and the surrounding area. Material conduction was assumed to simulate the heat transfer inside the specimen. The stress fields in this step acted on the internal surface of the drilled hole similar to the first approach (IHDM-1).

**Figure 5:**
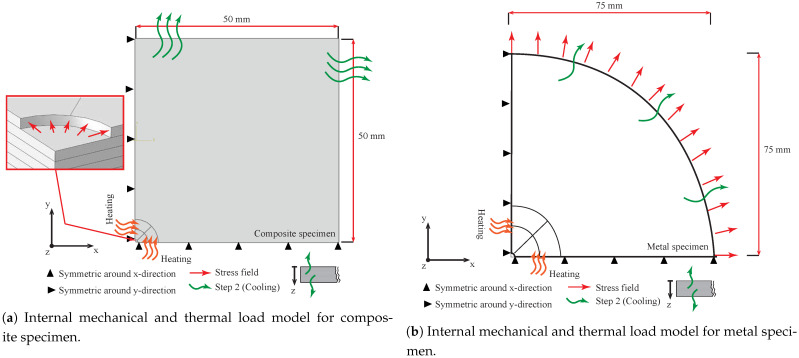
Internal mechanical and thermal load models.

A coupled temperature-displacement element type was defined for the entire specimen. The temperature of the specimen was experimentally measured around the hole after each drilling increment, as explained in Section 4.3. A similar temperature to the experimental measurements for each increment was assigned to the elements, which represented the volume underneath the strain gauge rosette. A temperature of 100 ${}^{\circ }$C and 80 ${}^{\circ }$C were assumed for the steel and CFRP workpieces, respectively. These high temperatures represented the material heating during the drilling process. A subsequent relaxation step was assumed to include the specimen cooling by subjecting it to natural convection. The heat convection was deactivated in the first step as it had an insignificant effect compared with conduction inside the specimen during the drilling procedure. The workpiece was left to cool to room temperature, while all the other loads were dismissed. A convection coefficient of 10 W/(m${}^{2}$ ${}^{\circ }$C) and a sink temperature of 20 ${}^{\circ }$C were assumed in this approach. In addition, the heat radiation was neglected in the entire analysis [31]. Subsequently, the strains were measured for each gauge as performed in the internal stress field approach. Figure 5 shows all the material thermal and mechanical constraints during the two steps. The composite and steel models were run 16 times each to conduct the 8 drilling increments.

## 4. Materials and Methods

This section describes the material specifications included in the present study. The machining of the metal specimens along with the composite manufacturing process are also covered. Finally, the procedure of the RSs measurements is also reported.

### 4.1. Steel Machining

The data obtained in [31] were compared with the current IHDM measurements of RSs to determine the effectiveness of the proposed IHDM approaches. The metal samples and the cutting procedure were conducted similarly to that described in the literature [31] to verify the difference between the XRD and the IHDM measurements. Steel disks of AISI 1045 (170 HV) with a diameter of 150 mm and thickness of 30 mm were used to conduct the experiments. Table 1 reports the details of the AISI 1045 mechanical properties.

**Figure 6:**
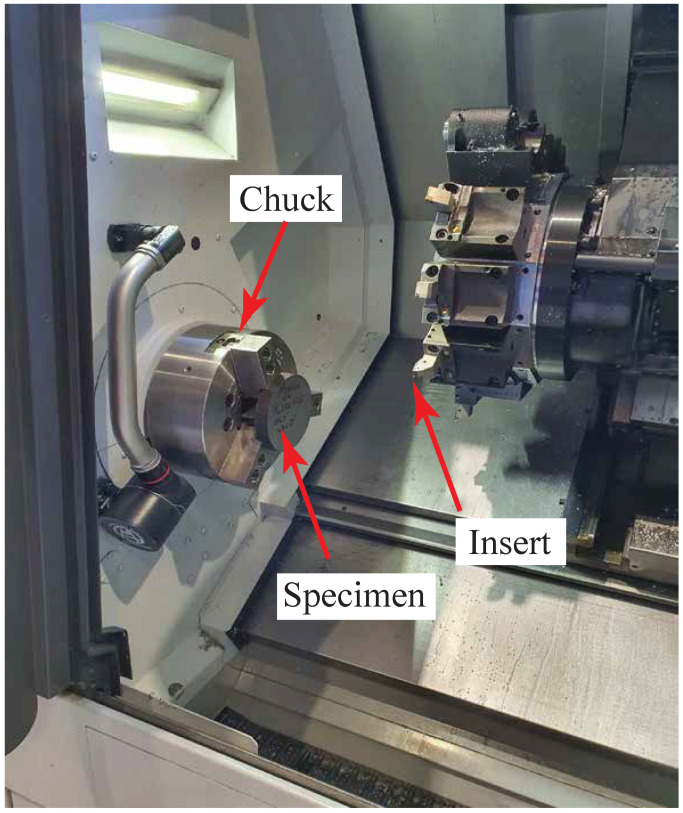
Metal specimen in CNC lathe.

Dry orthogonal cutting was conducted on the discs using the high-precision CNC lathe (DMG MORI NLX2500/1250). Figure 6 shows the metal specimen mounted in the lathe. Double-sided rhombic diamond inserts (DNGG 150401-SF) with zero rake angle were utilized to perform the cutting process. Two different cuts were applied; the first had a feed rate of 0.07 mm/rev, and the second was 0.14 mm/rev. The cuts were performed with a cutting speed of 100 m/min. The tests were replicated three times under the same conditions and a fresh insert was used for each experiment. All the machined samples were provided for the RSs measurements, as will be explained in Section 4.3.

### 4.2. Composite Manufacturing

The proposed models were also used to determine RSs inside the composite laminates.The composite prepreg tows (T700/2500) [33] from Torayca America, Inc. were used to build the composite samples in the current experiments. The material properties at ambient temperature are shown in Table 2.

**Figure 7:**
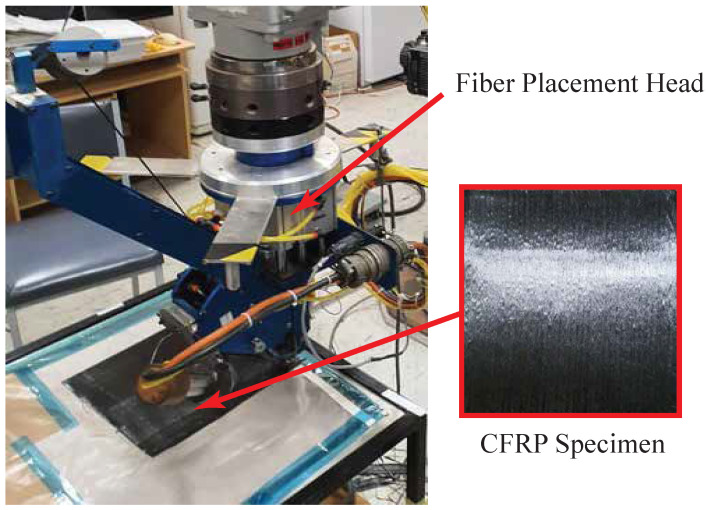
Composite manufacturing using the robotic fiber placement process.

The composite samples were manufactured using the automated manufacturing facility in the RMRL Lab, Monash University. The RFP process [34,35] was applied to fabricate the CFRP samples. An industrial manipulator (Yaskawa sk120), as well as a fully automated fiber placement head, were utilized to prepare the composite samples. Figure 7 shows the robotic manufacturing of unidirectional CFRP samples. The samples were manufactured under the same process conditions. The compression force, force direction, heating temperature, heat rate, and placement speed were all controlled and maintained using a Modicon programmable logic controller (PLC). The automated manufacturing provided a fully controlled and precise fabrication process that secured identical characteristics of all the manufactured samples. All the produced samples were vacuum packed and subsequently placed into an autoclave with controlled pressure and temperature until they became fully cured. All the samples were allowed a cooldown period before measuring the developed RSs. Each sample included 20 unidirectional layers, with 3 mm-thickness and 200 mm for both width and length.

### 4.3. Stresses Evaluation Procedure

**Figure 8:**
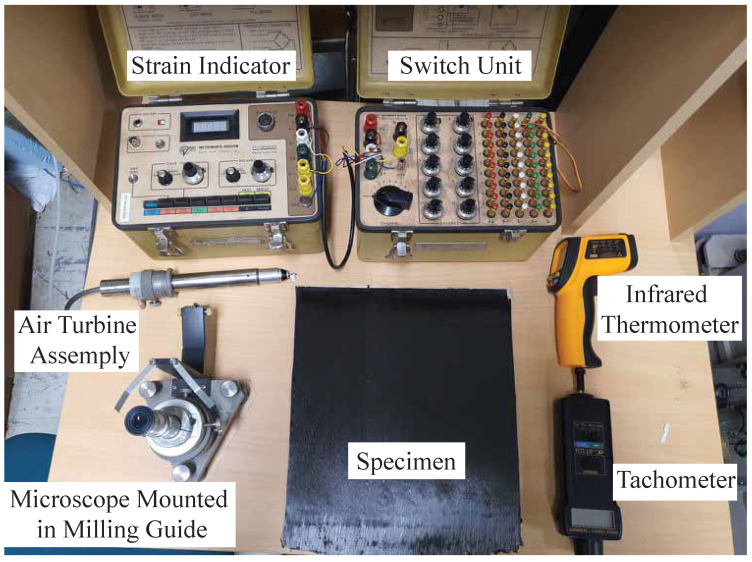
Incremental hole-drilling method (IHDM) equipment.

All the suggested approaches were applied to calibrate the IHDM to provide accurate measurements. The IHDM was conducted through two different operations. First, the specimen was drilled using a high-speed drilling setup. Second, the strains were measured around the drilled hole in different directions using a sensory-based measurement system. The equipment used to perform the IHDM is presented in Figure 8. A tiny hole was drilled by an air turbine connected to a carbide cutter. The drilling speed and feed rate were selected based on the optimized data obtained in [38]. A speed of 20,000 RPM and a feed rate of 10 μm/s were adopted in this work. The speed was adjusted through the pressure of the air supply to the turbine assembly. The speed was measured using a tachometer. The feed rate was adapted by a micrometer assembled on a milling guide. A locking collar was placed on the top of the assembly to prevent any back motion of the drilling tool.

**Figure 9:**
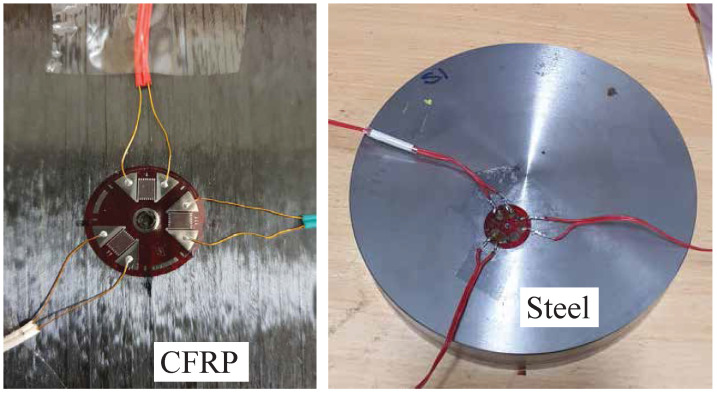
Measuring strains in composite and metal samples.

The strains were measured using a rosette strain gauge of 45${}^{\circ }$ type (FRS-3-11-1 LJB). The individual gauges had 3 mm-length, 120 Ω-resistance, and a center diameter of 17.5 mm. The gauges were bonded to the surface of the samples, as shown in Figure 9. The directional strains were measured after each increment in three directions; 0${}^{\circ }$, 135${}^{\circ }$, and −90${}^{\circ }$. A period of 2 min was kept between the end of drilling and the start of measuring the strains to allow cooldown of the specimen. The temperature was measured around the drilled hole after each increment using an infrared thermometer. The strains were monitored through a digital strain indicator (Vishay P-3500) connected to a switch/balance unit. The measured strains through the gauges were sensitive to the location of the hole with respect to the center of the gauges. Therefore, an optical microscope was used to align the drill bit with the center of the gauges.

## 5. Results

All the stresses measured in the machined steel samples and the manufactured composites are described in the current section. The results obtained using the proposed methodologies are investigated and compared with the XRD measurements.

### 5.1. Residual Stresses in Machined Steel

**Figure 10:**
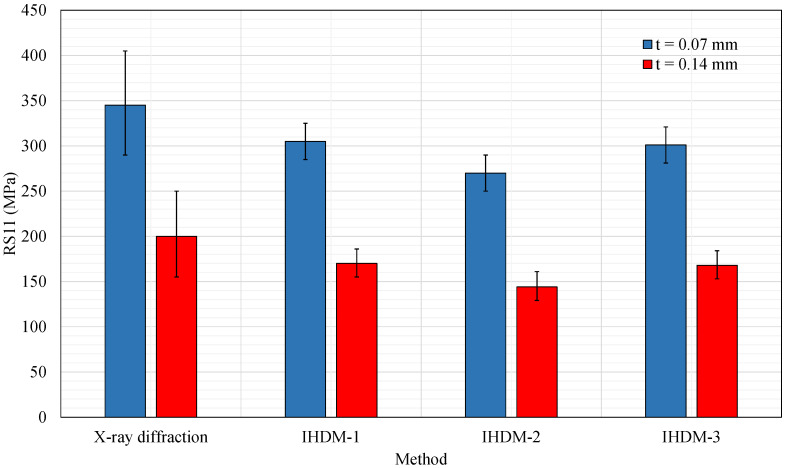
Steel surface residual stresses in cutting direction for two different feed rates.

This part describes the validation process of using the IHDM to measure the RSs. The data obtained in [31] showed the measurements of RSs using the XRD method. The RSs were measured on the surface of machined AISI 1045 steel disks in the cutting direction. The XRD was used as a reference for the current work as it had the potential to measure the RSs precisely inside the metal structures. In the present work, the IHDM was applied with the three calibration approaches to determine the stresses induced in the steel disks which were machined under the same conditions as [31]. Figure 10 shows the surface RSs in the cutting directions (RS11) obtained from four different techniques. The standard deviations are also presented in the figure. The RSs were underestimated using all the approaches of IHDM compared with the XRD technique. The metal cutting in the present work was performed with inserts of 100 μm-edge-radius (i.e., the minimum available edge-radius of the diamond cutter). However, the prepared specimens for the XRD measurements were machined with sharper tools that had an edge-radius of 22 μm. Thus, the sharp tools would induce more tensile RSs, which agrees with [31]. Another reason for the underestimation of the IHDM models is that the XRD measured the surface RSs in a spot of 3 mm. However, the IHDM was performed with a smaller spot size (i.e., the hole diameter) of 2 mm. The decrease in the area of measurement caused a decrease in the RSs values provided by the IHDM. The IHDM detected lower RSs at a higher feed rate (chip thickness (t) = 0.14 mm) similar to the XRD, and this also agrees with the findings in [31]. The standard deviation in the case of all the IHDM measurements was very low compared with the XRD, which reflects the high accuracy of the IHDM.

**Figure 11:**
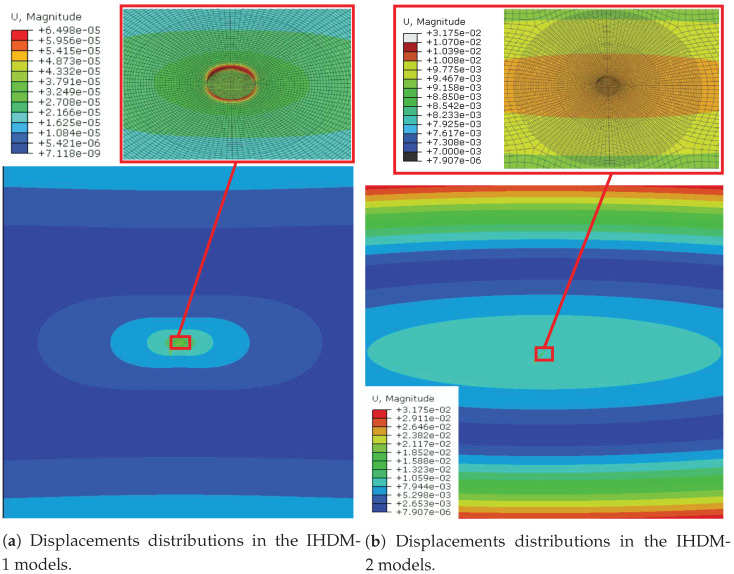
Displacements’ distributions on the specimen’s surface.

The first approach (IHDM-1) estimated the closest values to the XRD measurements for the two feed rates, with a difference of 40 MPa and 30 MPa for the t = 0.07 mm and t = 0.14 mm, respectively. The second approach (IHDM-2) estimated lower values than IHDM-1, as the difference reached a maximum of 11%. This model was conducted using external loads that acted on the outer borders of the specimens, which was different from the real experiments. When subjecting the loads to the outer surfaces of the specimen, the area surrounding the hole, i.e., where the gauges were located, experienced moderate variations in displacements across the strain gauges’ length as shown in Figure 11. The displacement distribution was different in the IHDM-1, as it encountered considerable variation in the displacements profile around the hole. Nevertheless, the strain values in IHDM-2 were higher than the corresponding values in IHDM-1, which overcame the effect of the lower variations in the displacements profiles. The rise in the displacement values was expected due to the increase in the area subjected to the loads, as the outer surfaces have larger areas than the internal surface of the drilled hole. Thus, the calibration coefficients were higher in IHDM-2, and accordingly, lower stresses were calculated through the theoretical approach described in Section 2.1.

The values in the third approach (IHDM-3) were very close to the IHDM-1 with a slight underestimation in this model. Including the temperature effect after the drilling operations increased the displacement values caused by the same loads. The increase in the values of the calibration coefficients resulted in a decrease in the calculated RSs. Yet, this difference was insignificant as it was less than 1% in all the cases. The difference between the IHDM-1 and IHDM-2 was insignificant due to the low effect of the drilling on the sample temperatures. The heating of the specimen was not enough to cause a large difference between the two models, as the temperature of the area around the hole was below 100 ${}^{\circ }$C. In other circumstances, with different feed rates and drilling speeds, this effect may increase and cause a significant decrease in the calculated stresses.

We suggest that the IHDM-3 is the best approach to describe the real experiments; however, it was not the closest one to the XRD values. This would be different if the machining occurred with the same edge-radius of the inserts and the same spot size of the measurements (i.e., an increase in all the values of the IHDM models would be detected). Therefore, the IHDM-3 would provide more accurate results than the other models.

### 5.2. Residual Stresses in Composite Samples

**Figure 12:**
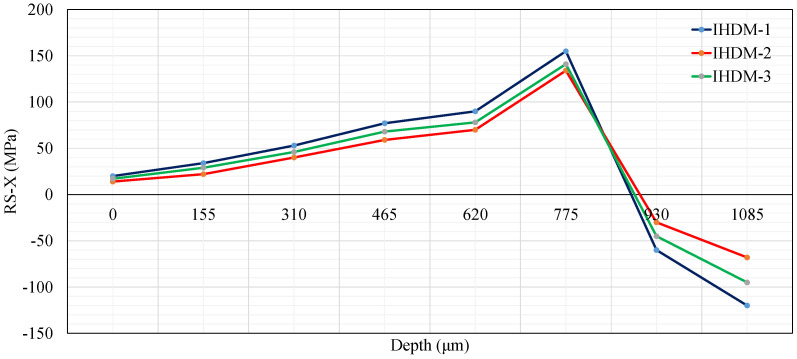
CFRP residual stresses in the X-direction.

A further three models were constructed in order to investigate the differences between the three IHDM approaches in evaluating the surface and in-depth RSs of the CFRP samples. The calibration process using the three approaches was applied to the strain measurements of the identical samples. Figure 12 shows the RSs measured in the fiber direction (i.e., X-direction) using the IHDM through the three different calibration techniques. As reported in [21,39], the IHDM could measure the stresses accurately close to the surface; however, the accuracy decreased when measuring the stresses in the deeper layers, especially when passing a specific threshold, which depends on the process conditions and the specimen geometry. Accordingly, the stresses were measured only across the first 8 layers. An increment was assigned to each layer. The three models produced the same trend of the RSs with an increase in the tensile stresses in the first 6 layers, while compression stresses were detected when reaching close to the middle layers (layers 7 and 8). This is because the samples were fabricated using the RFP technique, subjecting each layer to heat flux and compression forces [40]. The deeper layers were restricted by the surface layers. Subsequently, when the material was left to relax, the tensile stresses were developed in the outer layers, and compression stresses were generated in the internal laminate.

The differences in RSs values between the three approaches were insignificant at the surface layer, which is similar to the findings in Section 5.1. However, when moving towards the specimen’s center, the differences increase between the three models and reach a maximum of 43% (52 MPa) between the IHDM-1 and IHDM-2 in the last layer. It is believed that the rise in the differences occurred because each new increment added more difference to the contributions of the previous layers, as mentioned in Section 5.1. This would maximize the differences between the three approaches compared with the previous increments.

**Figure 13:**
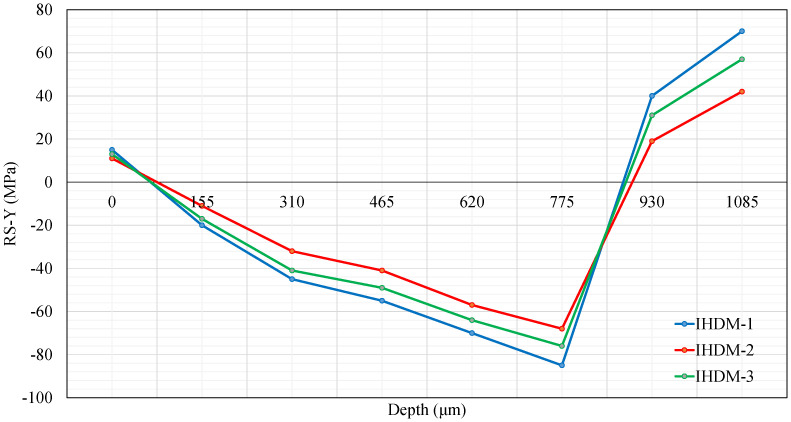
CFRP residual stresses in the Y-direction.

Different from the RSs in the X-direction, compression stresses in the Y-direction were developed in the layers close to the surface and turned into tensile stresses in the last two layers, which agrees with [20,24]. Figure 13 shows the stresses induced in Y-direction. The stresses measured using IHDM-3 were still close to the values of IHDM-1 within the 8 layers. The values of stresses for the three approaches diverged when measuring the stresses in the internal layers. The difference increased, again, adding to the contributions of the previous layers. The RSs reached a peak in layer 6, similar to the stresses in the X-direction. However, the largest difference was detected in layer 8 between IHDM-1 and IHDM-3.

Generally, the three approaches were close in the measured RSs values with a noticeable increase in the differences in the internal layers. The IHDM-3 estimated closer values to the basic approach (i.e., IHDM-1) in all the layers. Therefore, the heat due to drilling had an insignificant effect on the CFRP properties under the current circumstances. As mentioned in [41], the temperature had a considerable effect on the mechanical properties of the thermoset composites only if it was higher than the resin glass transition temperature. In the present analysis, the glass transition temperature was 145 ${}^{\circ }$C, which was much higher than the maximum temperature of the specimen during drilling. In contrast, IHDM-2 estimated higher deviations from the values provided by IHDM-1. Overall, the three models produced the same type of stresses in each direction and also the same trend (either rise or reduction) in the stresses between the layers.

Table 3 shows the surface RSs estimated through the three approaches of IHDM in the machined isotropic material (i.e., AISI 1045) and the automated manufacturing of orthotropic composites (i.e., CFRP), simultaneously. As presented in the table, the differences between the three approaches were higher for the CFRP, which reached 30% between IHDM-1 and IHDM-2 in the X-direction, while the steel samples had fewer differences, with a maximum of 15% between the first two approaches. These high differences in the composite specimen occurred because the load acts on a larger area (i.e., two rectangles on two sides) which triggered more stresses in the IHDM-2. The steel specimen was circular; thus, the external loads acted on less area (i.e., closer to the internal surface area of the hole). Additionally, there were no clear differences detected between IHDM-1 and IHDM-3 in both materials as the temperature effect was insignificant under the current experimental conditions.

## 6. Conclusions

The current study was performed to investigate the role of the calibration process in the IHDM. Three different calibration methods were established and compared. First, the numerical model was constructed based on applying the loads to the internal surfaces of the drilled hole. Second, the model was modified to include external loads acting on the borders of the specimen. Third, the thermal effect of the drilling operations, as well as the mechanical loads’ effect on the workpiece, was included. In all the approaches, the displacement field was investigated in the area of the strain gauges and surrounding the hole. The measuring techniques were applied to AISI 1045 as well as CFRP. The orthogonal cutting of the steel specimen was conducted with two feed rates, and the stresses were measured inside the machined specimens. The obtained results of the steel specimens were validated by comparing them with XRD measurements. Under the current conditions, the results showed no clear differences between the values of the basic model (IHDM-1) and the IHDM-3, which assumed pure internal mechanical loads and combined mechanical and thermal loads, respectively. In contrast, an underestimation of the stresses was detected in the approach that included external loads (IHDM-2). It is important to note that the machining of the steel specimen with a cutting tool that had a higher edge radius induced less tensile stresses compared to the sharper tool. Additionally, the IHDM showed a better accuracy compared with the XRD in estimating the surface RSs.

On the other hand, the composite components were fabricated and cured based on the automated manufacturing technique, and subsequently, the generated stresses were measured with the IHDM. The three approaches had less effect on the surface RSs; however, the differences increased when measuring the stresses inside the internal layers. The compression and the heat applied to the outer layers induced extra stresses inside the internal layers. Hence, higher values of the RSs were estimated in the deeper layers through all the approaches. In general, the third approach is believed to be the most accurate method, because it considers all the effects of the mechanical and thermal loads on the specimens.

Further studies will be conducted to investigate the differences between the proposed approaches on multi-axes laminates as well as hybrid composites, thus aiming to reach the best estimation of RSs inside the composite materials.

## Figures and Tables

**Table 1:** AISI 1045 mechanical properties [32].

Property	Value
Density (g/cm${}^{3}$)	7.87
Young’s modulus *E* (GPa)	200
Shear modulus *G* (GPa)	80
Poisson’s ratio ν	0.29
Thermal conductivity λ (W/m.K)	51.9
Surface roughness Ra (μm)	4
Coefficient of thermal expansion α (× 10${}^{-6}$ ${}^{\circ }$C${}^{-1}$)	11.2
Ambient temperature $T_{o}$ (${}^{\circ }$C)	25
Temperature due to drilling effect $T_{1}$ (${}^{\circ }$C)	100

**Table 2:** CFRP (T700/2500) mechanical properties [36,37].

Property	Value
Density (g/cm${}^{3}$)	1.525
Longitudinal Young’s modulus $E_{11}$ (GPa)	130.1
Transverse Young’s modulus $E_{22}$ (GPa)	8.03
Normal Young’s modulus $E_{33}$ (GPa)	9.1
In-plane shear modulus $G_{12}=G_{13}$ (GPa)	5.22
Out-of-plane shear modulus $G_{23}$ (GPa)	3.1
Poisson’s ratio $\nu_{12}=\nu_{13}$	0.31
Poisson’s ratio $\nu_{23}$	0.49
Longitudinal thermal conductivity $\lambda_{11}$ (W/m.K)	7
Transverse thermal conductivity $\lambda_{22}$ (W/m.K)	0.8
Out-of-plane thermal conductivity $\lambda_{33}$ (W/m.K)	0.8
Longitudinal coefficient of thermal expansion $\alpha_{11}$ (× 10${}^{-6}$ ${}^{\circ }$C${}^{-1}$)	0.3
Transverse coefficient of thermal expansion $\alpha_{22}$ (× 10${}^{-6}$ ${}^{\circ }$C${}^{-1}$)	36.5
Out-of-plane coefficient of thermal expansion $\alpha_{33}$ (× 10${}^{-6}$ ${}^{\circ }$C${}^{-1}$)	36.5
Surface roughness Ra (μm)	3.8
Ambient temperature $T_{o}$ (${}^{o}C$)	25
Temperature due to drilling effect $T_{1}$ (${}^{o}C$)	80

**Table 3:** Surface residual stresses.

Method	AISI 1045 (t = 0.07 mm)	AISI 1045 (t = 0.14 mm)	CFRP(RS-X)	CFRP(RS-Y)
IHDM-1	305 MPa	170 MPa	20 MPa	15 MPa
IHDM-2	270 MPa	144 MPa	14 MPa	11 MPa
IHDM-3	301 MPa	168 MPa	17 MPa	13 MPa

## Data Availability

Not applicable.

## References

[1] Puymbroeck E.V., Nagy W., Schotte K., Ul-Abdin Z., Backer H.D. (2019). Determination of Residual Welding Stresses in a Steel Bridge Component by Finite Element Modeling of the Incremental Hole-Drilling Method. Appl. Sci..

[2] Pagliaro P., Prime M.B., Robinson J.S., Clausen B., Swenson H., Steinzig M., Zuccarello B. (2011). Measuring Inaccessible Residual Stresses Using Multiple Methods and Superposition. Exp. Mech..

[3] Chang P.H., Teng T.L. (2004). Numerical and experimental investigations on the residual stresses of the butt-welded joints. Comput. Mater. Sci..

[4] Frankel J., Abbate A., Scholz W. (1993). The effect of residual stresses on hardness measurements. Exp. Mech..

[5] Crecraft D.I. (1967). The measurement of applied and residual stresses in metals using ultrasonic waves. J. Sound Vib..

[6] Lee J., Jeong S., Lee Y.J., Sim S.H. (2019). Stress Estimation Using Digital Image Correlation with Compensation of Camera Motion-Induced Error. Sensors.

[7] Liaw H.C., Shirinzadeh B., Smith J. (2007). Enhanced sliding mode motion tracking control of piezoelectric actuators. Sens. Act. A Phys..

[8] Krishnamurthy S., Badcock R.A., Machavaram V.R., Fernando G.F. (2016). Monitoring Pre-Stressed Composites Using Optical Fibre Sensors. Sensors.

[9] Wei W., Shirinzadeh B., Nowell R., Ghafarian M., Ammar M.M.A., Shen T. (2021). Enhancing Solid State LiDAR Mapping with a 2D Spinning LiDAR in Urban Scenario SLAM on Ground Vehicles. Sensors.

[10] Chen C., Wu Q., Xiong K., Zhai H., Yoshikawa N., Wang R. (2020). Hybrid Temperature and Stress Monitoring of Woven Fabric Thermoplastic Composite Using Fiber Bragg Grating Based Sensing Technique. Sensors.

[11] Gong S., Schwalb W., Wang Y., Chen Y., Tang Y., Si J., Shirinzadeh B., Cheng W. (2014). A wearable and highly sensitive pressure sensor with ultrathin gold nanowires. Nat. Commun..

[12] Liang K., Angelopoulos S., Lepipas G., Tsarabaris P., Ktena A., Bi X., Hristoforou E. (2019). Sensor to Monitor Localized Stresses on Steel Surfaces Using the Magnetostrictive Delay Line Technique. Sensors.

[13] Schajer G.S. (1988). Measurement of non-uniform residual stresses using the hole drilling method—Part I: Stress calculation procedure. J. Eng. Mater. Technol..

[14] Blödorn R., Viotti M.R., Schroeter R.B., Albertazzi A. (2015). Analysis of Blind-Holes Applied in the Hole-Drilling Method for Residual Stress Measurements. Exp. Mech..

[15] Fitzpatrick M.E., Fry A.T., Holdway P., Kandil F.A., Shackleton J., Suominen L. (2005). Determination of Residual Stresses by X-ray Diffraction. Measurement Good Practice Guide No. 52. https://eprintspublications.npl.co.uk/2391/.

[16] Jiang W., Chen W., Woo W., Tu S.T., Zhang X.C., Em V. (2018). Effects of low-temperature transformation and transformation-induced plasticity on weld residual stresses: Numerical study and neutron diffraction measurement. Mater. Des..

[17] Kotobi M., Honarpisheh M. (2016). Uncertainty analysis of residual stresses measured by slitting method in equal-channel angular rolled Al-1060 strips. J. Strain Anal. Eng. Des..

[18] Bartlett J.L., Li X. (2019). An overview of residual stresses in metal powder bed fusion. Addit. Manuf..

[19] Valentini E., Beghini M., Bertini L., Santus C., Benedetti M. (2011). Procedure to perform a validated incremental hole drilling measurement: Application to shot peening residual stresses. Strain.

[20] Held E., Schuster S., Gibmeier J. (2014). Incremental hole-drilling method vs. thin components: A simple correction approach. J. Adv. Mater. Res..

[21] ASTM E837-13a (2013). Standard Test Method for Determining Residual Stresses by the Hole-Drilling Strain-Gage Method. https://scholar.google.com.sg/scholar?hl=en&as_sdt=0%2C5&q=ASTM+E837-13a.+Standard+Test+Method+for+Determining+Residual+Stresses+by+the+Hole-Drilling+Strain-Gage+Metho+2013d%3B&btnG=.

[22] Blödorn R., Bonomo L.A., Viotti M.R., Schroeter R.B., Albertazzi A. (2017). Calibration Coefficients Determination Through Fem Simulations for the Hole-Drilling Method Considering the Real Hole Geometry. Exp. Tech..

[23] Akbari S., Taheri-Behrooz F., Shokrieh M.M. (2014). Characterization of residual stresses in a thin-walled filament wound carbon/epoxy ring using incremental hole drilling method. Compos. Sci. Technol..

[24] Sicot O., Gong X.L., Cherouat A., Lu J. (2003). Determination of residual stress in composite laminates using the incremental hole-drilling method. J. Compos. Mater..

[25] Ghasemi A.R., Mohammadi M.M. (2016). Residual stress measurement of fiber metal laminates using incremental hole-drilling technique in consideration of the integral method. Int. J. Mech. Sci..

[26] Simon N., Mrotzek T., Gibmeier J. (2018). Reliable residual stress analysis for thin metal sheets by incremental hole drilling. Mater. Perform. Charact..

[27] Nasr M.N., Ammar M.M. (2017). An Evaluation of Different Damage Models when Simulating the Cutting Process Using FEM. Procedia CIRP.

[28] Shirinzadeh B., Alici G., Foong C.W., Cassidy G. (2004). Fabrication process of open surfaces by robotic fibre placement. Robot. Comput.-Integr. Manuf..

[29] Zhao P., Shirinzadeh B., Shi Y., Cheuk S., Clark L. (2018). Multi-pass layup process for thermoplastic composites using robotic fiber placement. Robot. -Comput.-Integr. Manuf..

[30] Sicot O., Gong X., Cherouat A., Lu J. (2004). Influence of experimental parameters on determination of residual stress using the incremental hole-drilling method. Compos. Sci. Technol..

[31] Nasr M.N.A. (2015). Effects of sequential cuts on residual stresses when orthogonal cutting steel AISI 1045. Procedia CIRP.

[32] AISI 1045 Carbon Steel (UNS G10450). https://www.azom.com/article.aspx?ArticleID=9153.

[33] Zhao P., Shirinzadeh B., Shi Y., Cheuk S., Clark L. (2017). Improved uniform degree of multi-layer interlaminar bonding strength for composite laminate. J. Reinf. Plast. Compos..

[34] Ammar M.M.A., Shirinzadeh B., Zhao P., Shi Y. (2021). An approach for damage initiation and propagation in metal and carbon fiber hybrid composites manufactured by robotic fiber placement. Compos. Struct..

[35] Ammar M.M.A., Shirinzadeh B., Zhao P., Shi Y. (2020). Developing a Trajectory Planning for Curved-Contoured Surfaces for Use by 8-DoF Workcell in Robotic Fibre Placement. Conference Series: Materials Science and Engineering.

[36] Takeda S., Minakuchi S., Okabe Y., Takeda N. (2005). Delamination monitoring of laminated composites subjected to low-velocity impact using small-diameter FBG sensors. Compos. Part A Appl. Sci. Manuf..

[37] Viorel A., Nicolae C., Andreea B., Mircea G., Ştefan S. (2010). On the use of infrared thermography as NDT of aerospace materials. INCAS BULLETIN.

[38] Nobre J.P., Stiffel J.H., Van Paepegem W., Nau A., Batista A.C., Marques M.J., Scholtes B. (2011). Quantifying the drilling effect during the application of incremental hole-drilling technique in laminate composites. Mater. Sci. Forum.

[39] Schajer G.S. (1988). Measurement of non-uniform residual stresses using the hole- drilling method. part ii-practical application of the integral method. J. Eng. Mater. Technol. Trans. ASME.

[40] Aized T., Shirinzadeh B. (2011). Robotic fiber placement process analysis and optimization using response surface method. Int. J. Adv. Manuf. Technol..

[41] Barker A.J., Vangerko H. (1983). Temperature dependence of elastic constants of CFRP. Composites.

